# High throughput in situ metagenomic measurement of bacterial replication at ultra-low sequencing coverage

**DOI:** 10.1038/s41467-018-07240-8

**Published:** 2018-11-23

**Authors:** Akintunde Emiola, Julia Oh

**Affiliations:** The Jackson Laboratory for Genomic Medicine, Farmington, 06032 CT USA

## Abstract

We developed Growth Rate InDex (GRiD) for estimating in situ growth rates of ultra-low coverage (>0.2×) and de novo-assembled metagenomes. Applying GRiD to human and environmental metagenomic datasets to demonstrate its versatility, we uncovered new associations with previously uncharacterized bacteria whose growth rates were associated with several disease characteristics or environmental interactions. In addition, with GRiD-MG (metagenomic), a high-throughput implementation of GRiD, we estimated growth dynamics of 1756 bacteria species from a healthy skin metagenomic dataset and identified a new *Staphylococcus-Corynebacterium* antagonism likely mediated by antimicrobial production in the skin. GRiD-MG significantly increases the ability to extract growth rate inferences from complex metagenomic data with minimal input from the user.

## Abstract (web summary)

Bacterial growth rate can change under different environmental conditions and reveals important biological and ecological information. Here, the authors develop GRiD, a bioinformatics tool for high-throughput estimation of growth rate using low coverage metagenomic data.

## Introduction

Metagenomic shotgun sequencing has emerged as a powerful tool to interrogate the composition and function of complex microbial communities^1,2^. Yet such characterizations do not reflect the dynamic nature of a complex microbial community in which microbial growth rate can change under different environmental conditions or during disease. Bacterial growth rate measurements can reveal the contributions of viable populations to overall microbial abundance, providing insight into microbes that may be the active contributors to the community phenotype. Moreover, new insights into antagonistic interspecies interactions can be identified by estimating ratios of rapidly growing vs. dead/stationary cells.

Korem and colleagues^3^ first developed an estimation of bacterial growth rate from metagenomic shotgun data using peak-to-trough ratio (PTR). This is based on the principle that most bacteria harbor a single circular chromosome that is replicated bi-directionally commencing from the origin of replication (*ori*) to the terminus (*ter*) region^4^. Therefore, rapidly dividing cells will have higher read coverage and thus, higher PTR at *ori* vs. *ter* (Supplementary Fig. 1A). However, this method relies on a closed circular reference genome and is therefore inadequate for the vast majority of microbial genomes.

A similar approach, iRep^5^, was recently developed to estimate growth rate using draft genomes. iRep maps metagenomic reads to a draft genome to calculate coverage, then orders the contigs from highest to lowest coverage to approximate a PTR-like distribution in the absence of a complete genome. Very high and very low coverage regions are excluded to reduce noise from strain variation or highly conserved regions. A linear regression model is then used to deduce if a population is replicating. iRep’s key limitation is a requirement for >5× coverage, which on average, represents fewer than 5% of genomes in human microbial communities, such as the skin (Supplementary Fig. 1B).

Finally, both methods rely on identification and mapping of reads to a genome of interest on per-species basis. Given that many microbial communities contain hundreds of species, both PTR and iRep can be burdensome to scale. Moreover, if species of interest are selected based on relative abundance within a community, growth analyses of some biologically relevant microbes may be excluded, no/poor correlation has been observed between relative abundance and growth rate^3^.

Because of these restrictions, both methods have limited potential for real world metagenomes. Microbial communities vary in biomass, microbial complexity (the diversity of genomes present in an ecosystem), and population composition, with varying numbers of low-abundance bacteria. These factors significantly affect metagenomic assembly and analysis. This is important because improvements in metagenomic binning has made it possible to identify previously uncharacterized microbes from complex microbial communities. However, most of these reconstructed genomes are fragmented and only partially cover the genome. Moreover, complex metagenomes often contain closely related species and strains. Neither approach provide robust error estimates to account for noise or ambiguous read mapping. Being able to accurately and systematically estimate growth dynamics of microbes in a complex community would provide new insights into microbial interactions and disease associations.

Here, we developed Growth Rate Index (GRiD) for the measurement of microbial growth rate from complete/draft genomes and metagenomic bins at ultra-low sequencing coverage (0.2×, which is roughly equivalent to 0.05% relative abundance of a 2.5 Mbp genome from a metagenomic sample of 100 bp x 10 million reads). GRiD can be applied to a specific genome of interest, or can be utilized in a high-throughput mode for which prior knowledge of microbial composition and coverage is not required.

## Results and Discussion

### Overview of GRiD

**Fig. 1:**
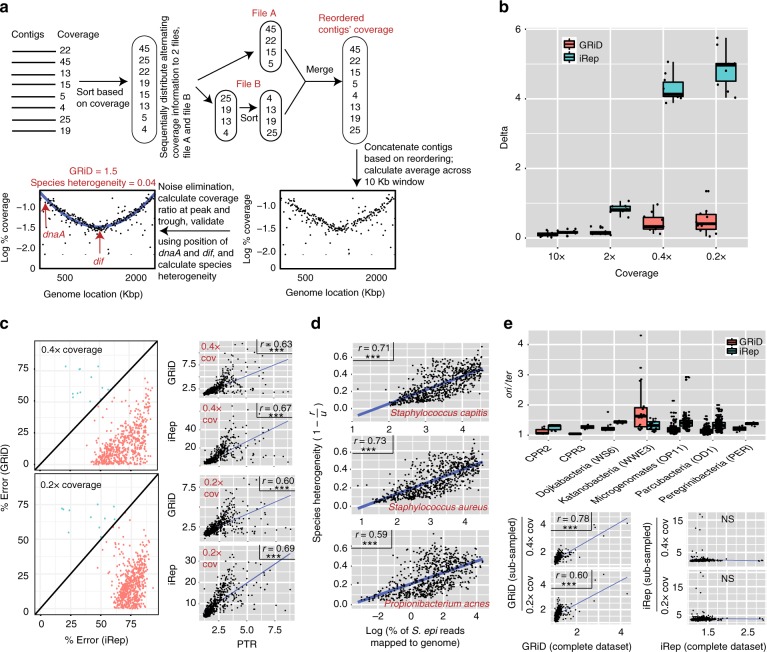
In situ growth estimate from ultra-low coverage bacteria. **a** The GRiD approach. Contigs are re-ordered to produce a pattern whereby low coverage contigs potentially containing *ter* are located near the mid-region of the genome, while high-coverage contigs potentially harboring *ori* are located at either extremes of the genome. GRiD values correspond to the ratio of coverage at the peak (*ori*) and trough (*ter*) regions. **b** Growth rate reproducibility between GRiD and iRep using reads obtained from pure cultures of *Staphylococcus epidermidis* and *Corynebacterium simulans*. The boxplot shows the difference (delta) in growth estimates before and after reads were subsampled to lower coverage. To avoid bias, only unrefined GRiD values (see methods) were used for comparison with iRep. **c** Error rate comparison between GRiD and iRep from a skin metagenomic dataset using *S. epidermidis* reference genome. PTR was calculated using a closed circular reference genome while GRiD and iRep were calculated using the same reference genome, but fragmented into 100kb fragments and reshuffled. For samples with genome coverage > 0.2 (*n* = 588), mapped reads were subsampled to ultra-low coverage prior to GRiD and iRep estimations. Here, ${\mathrm{Percent}}\,{\mathrm{error}} = {\textstyle{{({\mathrm{max}}({\mathrm{predicted}},{\mathrm{real}})) - ({\mathrm{min}}({\mathrm{predicted}},{\mathrm{real}}))} \over {({\mathrm{max}}({\mathrm{predicted}},{\mathrm{real}}))}}} \times 100$, where “predicted” represent GRiD or iRep scores, and “real” is the PTR score. Unrefined GRiD values were used for comparison with iRep. The figure on the right shows Pearson correlation plots of GRiD and iRep with PTR. *** = *p* < 0.001. **d** Reads from a skin metagenomic dataset mapping to *S. epidermidis* were remapped to the respective genomes. Re-mapped reads are considered as ambiguous reads. The scatter plot shows the correlation (Spearman) between ambiguous reads and species heterogeneity (1 −*r*/*u*), where *r* = refined GRiD and *u* = unrefined GRiD (see Methods). *** = *p* < 0.001. **e** iRep and GRiD measurement for CPR genomes. The scatter plots below show Pearson correlation plots of GRiD and iRep estimates before and after subsampling to ultra-low coverage. *** = *p* < 0.001. Center lines in boxplots represent the median and the edges represent the first and third quartiles. Source data are provided as a Source Data file

GRiD calculates the coverage of all contigs of a reference genome or metagenomic bin in a given sample and sorts them from high to low coverage. The sorted contigs are then reordered to two groups, with the goal of placing an *ori-*containing contig at or near an arbitrary genome “start” and a *ter-*containing contig near the mid-region of a genome (Fig. 1a). This approach approximates a synthetic circular genome. Next, like both iRep and PTR, GRiD calculates coverage drops across a sliding 10 Kb window.

GRiD then calculates a “refined” growth value with additional statistical filters to reduce noise. After removing an initial set of outliers, a smoothing curve is fitted by a re-descending M estimator with Tukey’s biweight function^6^. This enables a local fit that is resistant to noise arising from species heterogeneity. Then, like PTR, GRiD values represent the coverage ratio of the peak and trough of the curve, with higher ratios representing faster growth rates. But because genomes with very low coverage can be prone to noise (which we define as peak and trough means with large residual errors), GRiD refines growth values. Here, GRiD chooses for the peak coverage value the lowest point of expected variation of the mean, while the upper point of variance of the mean is selected for the trough coverage value (Supplementary Fig. 1C). This refinement step markedly increases the reproducibility (i.e., reduced delta values) of growth estimates at ultra-low coverage levels (Supplementary Fig. 1D).

In contrast to both iRep and PTR, GRiD provides as standard output multiple confidence estimates, including confidence intervals by bootstrapping, estimates of error contributed by closely related species and strains in the community, and guidelines for growth rate correction. First, the GRiD algorithm accounts for uncertainty in contig ordering by subsampling mapped reads and re-calculating growth rate to derive a 95% confidence interval of growth estimates. Additionally, because metagenomic bins can be contaminated with contigs from other genomes, which may impact growth predictions in samples with high coverage of the contaminant, we introduced a quality control step using the position of the universal chromosome initiator replication gene (*dnaA*), and deletion-induced filamentation (*dif*) sequences (a conserved 28 bp sequence) across the genome, when available. In most bacterial genomes, *dnaA* is located in close proximity to the *ori*^7^ (Supplementary Fig. 1E), while replication typically terminates at/near *dif* sequences^8,9^. Therefore, accurate GRiD predictions in rapidly dividing cells should have *dnaA* and *dif* coverage similar to those of *ori* and *ter*, respectively (Fig. 1a). Finally, GRiD outputs a metric, called “species heterogeneity” which is an estimate of the degree to which closely related species contributes to variance in growth predictions, which would result from reads that are incorrectly assigned to the genome of interest.

### GRiD is accurate for draft genomes at ultra-low coverage

We tested our method using pure cultures of *Staphylococcus epidermidis* (NIHLM023) and *Corynebacterium simulans* (Wattiau) harvested at different time points during exponential growth (Supplementary Fig. 1F). To calculate GRiD and iRep, we utilized draft sequences of strains of the aforementioned species (*S. epidermidis* NIHLM023; *C. simulans* strain 1B08) and asked if initial estimates by both methods can be accurately reproduced when subsampled to ultra-low coverage levels. GRiD was highly reproducible compared to iRep’s low reproducibility (Fig. 1b).

To demonstrate real-world applications enabled by GRiD, we examined *S. epidermidis* growth rate in a longitudinal skin metagenomic dataset of 698 samples of varying depth and microbial diversity^10^. We chose *S. epidermidis* because of its ubiquity yet large relative abundance range in the skin. Moreover, it would provide a difficult scenario for growth rate prediction due to prevalent genetic strain variation^11^, which would increase noise. First, we benchmarked PTR using a closed reference genome, and then the same genome fragmented into 100kb fragments and reshuffled to mimic a draft genome for GRiD and iRep measurements. Reads mapping to *S. epidermidis* were subsampled to 0.4× and 0.2× coverage and subsequently used for GRiD and iRep estimates. iRep performed similarly to the PTR benchmark, but GRiD had a much lower percentage of error in comparison to iRep at both 0.4× and 0.2× coverage (Fig. 1c). To highlight the importance of accounting for ambiguous reads during growth estimation, reads mapping to *S. epidermidis* were re-mapped to *S. capitis, S. aureus*, and *Propionibacterium acnes* genomes to determine the proportion of multiple-mapping reads. Samples with increasing numbers of multi-mapped, ambiguous reads were significantly correlated with our metric of increasing species heterogeneity (Fig. 1d), which can increase uncertainty in growth rate estimation. For quality control, we found that a combination of *dnaA* coverage, *dif* coverage, and species heterogeneity can be used to ascertain the accuracy of growth predictions. Our findings suggest that growth rates are most accurate when *dnaA/ori* and *ter/dif* coverage ratios approach one, and species heterogeneity is low (<0.3, Supplementary Fig. 2A). Finally, we demonstrated that GRiD is effective even with highly fragmented genome/metagenomic bins – at 0.2× coverage, we found that GRiD requires ≤90 fragments/Mbp. At 1× coverage, GRiD is accurate even with >200 fragments/Mbp and 50% genome completeness (Supplementary Fig. 2B and C).

To demonstrate its versatility, we also benchmarked GRiD using uncultivated Candidate Phyla Radiation (CPR) genomes recovered from an environmental groundwater dataset^5^. CPR is a major subdivision within the domain Bacteria, and is characterized by small cells and genomes suggested to be symbionts^5^. Growth estimates using GRiD and iRep indicated these genomes are generally slow-growing (i.e., *ori/ter* *<* 1.5, Fig. 1e). GRiD and iRep values were uncorrelated. However, when we subsampled mapped reads to mimic ultra-low coverage, GRiD values were reproducible whereas iRep was not (Fig. 1e), underscoring iRep’s decreased performance as a function of coverage.

### GRiD uncovers new biology from metagenomic datasets

**Fig. 2:**
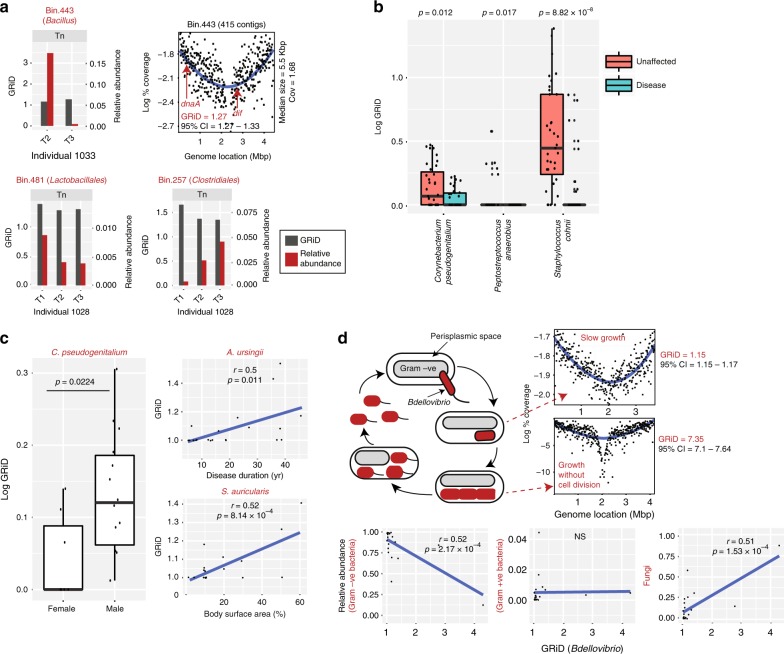
GRiD uncovers new biology from human and environmental communities. **a** Growth rate of rare uncharacterized bacteria over time from a skin metagenomic dataset. Uncharacterized bacteria were identified using de novo assembly of reads and metagenomic binning, with each bin representing a genome. The sampling interval between timepoints T1 and T2 is 10–30 months while sampling intervals between T2 and T3 are 5–10 weeks. Tn = toenail. **b** Growth rate of bacteria that were significantly different (*p* < 0.05, Wilcoxon rank-sum test) between unaffected and disease sites in a psoriasis skin metagenomic dataset. Center lines in boxplots represent the median and the edges represent the first and third quartiles. **c** Inter-individual bacterial growth differences and association with patient characteristics. Each data point in the scatterplots represents the average GRiD score of each patient. Statistical differences between population groups were determined using the Wilcoxon rank-sum test while Spearman correlation was utilized for correlation coefficient analyses. **d** Growth dynamics of *Bdellovibrio* species. Flagellated *Bdellovibrio* penetrates the outer membrane of Gram-negative bacteria into the periplasmic space (losing its flagella in the process), grows and elongates without cell division, resulting in a helical filament with multiple copies of the genome. As food supply from the host becomes exhausted, the filament divides by fission into multiple smaller cells and exits the host. GRiD plots represent examples of different growth phases in the life cycle of *Bdellovibrio* obtained from marine and sludge environmental samples. The scatter plots below show Spearman correlation between *Bdellovibrio* growth and relative abundance of different group of microbes. Each data point represents the average GRiD score per sample. Source data are provided as a Source Data file

We next investigated GRiD’s ability to provide new biological insights from metagenomic data by calculating growth dynamics of rare uncharacterized skin microbes reconstructed de novo from a large metagenomic dataset. We assembled reads, grouping contigs into “bins” approximating genomes based on co-abundance clustering and nucleotide composition^12^ (Supplementary Fig. 4). Using high quality bins (see Methods for criteria), we defined a genome bin as rare if it was present in fewer than 3% of samples. Three bins that could not be annotated to the species level met this threshold (bin.443, bin.481, and bin.257; Fig. 2a). These putative microbes were only present in a few individuals and had relatively stable growth rates over time despite fluctuations in relative abundance. Surprisingly, bin.257 appeared to stably colonize the toenail of one individual as indicated by increasing relative abundance over time while maintaining a constant growth rate (Fig. 2a). We noted that despite the low coverage (1.68×) and high fragmentation (415 contigs, median size = 5.5 Kb) of bin.443, GRiD contig reordering accurately positioned *dnaA* and *dif* sequences near the peak and trough regions respectively (Fig. 2a).

We further applied GRiD to a skin metagenomic dataset of individuals with psoriasis^13^, a long-lasting autoimmune disease characterized by patches of abnormal skin^14^, to see if GRiD could identify rapidly growing microbes or antagonistic interactions that could be associated with the disease state. Using draft genomes, the growth rate of three microbes differed between disease and unaffected sites (Fig. 2b). Strikingly, we found previously unidentified associations between microbial growth rate and patient characteristics (Fig. 2c and Supplementary Fig. 3A). For instance, *Corynebacterium pseudogenitalium* had higher average growth rates in male patients compared to females (Fig. 2c), in agreement with previous reports suggesting gender-specificity of some *Corynebacterium* species^15^. Inter-individual comparisons indicated that some microbes grew more rapidly in individuals with increased disease severity—Psoriasis Area Severity Index (PASI) and Body Surface Area (BSA)—were correlated with *Staphylococcus auricularis* growth, and longer disease duration with *Acinetobacter ursingii* (Fig. 2c and Supplementary Fig. 3A). These represent significant new biological insights that underscore the importance of the GRiD approach, as no associations were previously observed between microbial relative abundance and patient characteristics^13^.

Finally, we measured growth dynamics of uncharacterized *Bdellovibrio* species reconstructed from different environmental samples^16^. *Bdellovibrio* species are predators of Gram-negative bacteria and grow within the periplasmic space of their host^17^ (Fig. 2d). Although, we identified *Bdellovibrio* as generally slow-growing (consistent for endosymbionts, Supplementary Fig. 3B), certain microbes with very high GRiD values were identified in some samples (Fig. 2d). Such high GRiD values would be expected for fast growing microbes employing multi-fork replication. Notably, this is the first observation of a potential multi-fork replication in an endosymbiont. GRiD also corroborated known biology, identifying a negative correlation between *Bdellovibrio* growth and relative abundance of Gram-negative bacteria (Fig. 2d). Interestingly, *Bdellovibrio* growth was not correlated with abundance of Gram-positive bacteria, suggesting that microbial competition in non-human environmental communities is mainly restricted to Gram-negatives and Fungi (Fig. 2d).

### High-throughput estimation of bacterial growth

**Fig. 3:**
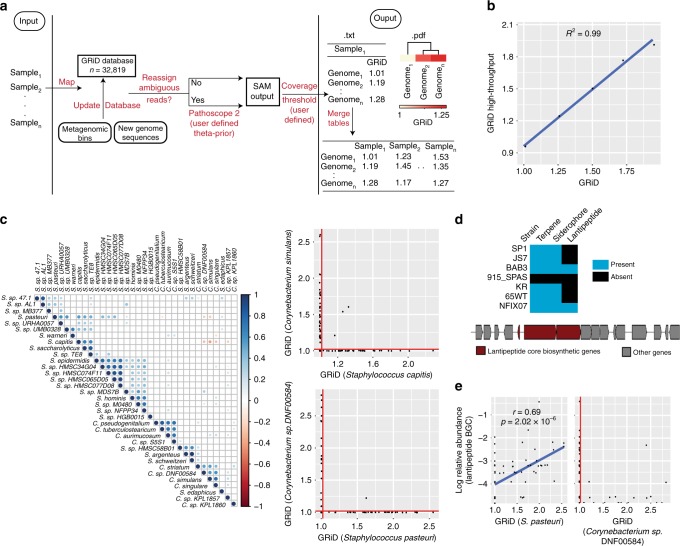
GRiD-MG for high throughput, multiplex estimations of growth rate. **a** Pipeline for GRiD-MG. Sample reads from a dataset are mapped to a GRiD-MG database. The database can be updated using metagenomic bins or newly sequenced genomes. Two output files are generated for every sample; a text file of genomes and their respective GRiD values, and a pdf file displaying heatmap of growth values with hierarchical clustering. **b** Correlation between GRiD-MG and GRiD values obtained using single isolate genomes. Mock reads were generated for 5 genomes for growth analyses. **c** Growth rate correlation between bacterial species in foot sites. Blue and red circles indicate positive and negative Spearman correlation respectively. Larger circles and darker colors indicate a higher correlation. The plot on the right represents competitive exclusion between *Staphylococcus* and *Corynebacterium* species. Microbes with GRiD score below 1.02 (vertical and horizontal red lines) were considered as non-replicating. **d** Biosynthetic gene clusters (BGC) identified in genome sequences of *S. pasteuri* strains retrieved from NCBI. The figure below shows a lantipeptide BGC identified in strain BAB3. All BGCs were predicted using antiSMASH^30^. **e** Spearman correlation between *S. pasteuri* growth rate and the relative abundance of its lantipeptide BGC. The figure on the right shows the effect of *S. pasteuri* lantipeptide BGC abundance on growth rate of *Corynebacterium sp*. GRiD scores below 1.02 (red vertical line) are considered as non-replicating. Source data are provided as a Source Data file

Having benchmarked our algorithm with diverse human and environmental datasets, we noted the significant value that could be derived from a systematic investigation of growth rate within a microbial community. Thus, we expanded GRiD’s utility as a high-throughput and systematic metagenomic (MG) analysis tool (GRiD-MG). In this case, growth rate can be estimated for most identified bacteria in a given sample passing coverage thresholds, without a requirement for prior knowledge of microbial composition or abundance. GRiD-MG maps samples from a dataset to a GRiD-MG database comprising of 32,819 representative bacterial species (Fig. 3a). The GRiD-MG database can be readily updated using metagenomic bins or custom genome sequences. Depending on user preferences, reads mapping to multiple genomes are re-assigned using Pathoscope 2.0^18^ and genomes with coverage values below the user-defined threshold are discarded (Fig. 3a). To ensure that growth estimates using GRiD-MG were concordant with results obtained using individual reference genomes, we synthesized mock reads for five genomes with varying coverage to mimic growing bacteria and calculated growth rate. Growth estimates were highly concordant in both cases (Fig. 3b).

We next applied GRiD-MG to our 698-sample metagenomic skin dataset and sought to identify potential antagonistic associations within the community. These samples were obtained from several body sites broadly classified based on skin site morphology as either dry, moist, oily, or foot sites^10^. Interestingly, growth antagonism was observed among several microbes that were site specific (Fig. 3c, Supplementary Fig. 3C – E). Notably, in foot sites, growth rates of *Staphylococcus capitis* and *Staphylococcus pasteuri* were antagonistic to several *Corynebacterium* species, and the observed antagonism was a likely scenario of competitive exclusion (Fig. 3c).

While *S. capitis* has previously been identified as a producer of antimicrobials capable of inhibiting numerous Gram-positive bacteria^19^, there is little information on *S. pasteuri*. We therefore hypothesized that *S. pasteuri* may produce antimicrobials to provide a competitive growth advantage within the skin community. We first screened all publicly available sequences of *S. pasteuri* strains for the presence of antimicrobial biosynthetic gene clusters (BGC) and identified two strains harboring a lantipeptide BGC (Fig. 3d). We then analyzed the presence of this BGC across all foot sites and found that its abundance was positively correlated with *S. pasteuri* growth, suggesting that its viability in foot sites is strongly dependent on antimicrobial production (Fig. 3e). Similarly, the occurrence of the lantipeptide BGC was also consistent with a putative inhibition of *Corynebacterium* (Fig. 3e). These data underscore the ability of GRiD-MG to uncover new associations and hypotheses based on large-scale analyses of growth rate dynamics within a microbial community.

In summary, GRiD and GRiD-MG provide a significant advance in their ability to systematically estimate bacterial growth rate using metagenomic data. GRiD is highly effective even at ultra-low sequencing coverage, making it particularly applicable to complex metagenomic datasets with draft quality and de novo assembled, uncultivated bacteria. However, it is important to note that the performance of many algorithms for metagenomic analyses can vary based on different input parameters. For example, using different assemblers or reference genomes with varying degrees of fragmentation with GRiD, iRep, or PTR can affect growth rate inferences. Therefore, best practice remains to broadly survey different predictions to avoid misrepresentations of the underlying biology. Here, we particularly recommend, where possible, to utilize a reference strain with the least fragmentation. Correspondingly, we constructed our default GRiD-MG database using the most complete set of representative strains as possible. This also supports GRiD’s particular attention to robustness and accuracy, as it provides multiple statistical estimates to minimize erroneous estimations due to noise that may arise from misassemblies in reconstructed genome bins or significant genetic strain variation that may be present in a sample. Finally, by examining growth rates in high-throughput, new associations between bacterial growth dynamics can be inferred, either in interspecies interactions or in new associations with, for example, host characteristics.

## Methods

### GRiD approach

GRiD measures the growth rate of uncharacterized bacteria or bacteria with draft quality genomes from metegenomic samples. GRiD is designed for use with a specific reference genome, or in a high-throughput manner for multiplex analysis of identified bacterial population present within a sample.

The GRiD algorithm is based on the principle that most bacteria harbor a circular chromosome that is replicated bi-directionally from the origin of replication (*ori*) to the terminus (*ter*) region^4^. Therefore, a rapidly dividing bacterium at any given time will have more copies of DNA close to the *ori* in comparison to the *ter*.

Reads from a metagenomic sample are first mapped to a given genome (including metagenomic bins or draft genome) and the mean coverage of each contig is calculated and contigs are sorted from high-to-low coverage. The sorted coverage information is then sequentially distributed alternatively between two temporary files, with one of the files re-sorted from low-to-high coverage (Fig. 1a). Both temporary files are then merged to produce a coverage pattern with high coverage contigs at both extremes, while low coverage contigs located mid-region. Using this sorted reordering, contigs are concatenated and coverage across each nucleotide is derived. GRiD then calculates coverage drops across a 10 Kb window and outlier points (i.e. values below Q1 – (1.5 × IQR) and above Q3 + (1.5 ×IQR); where Q1 and Q3 are the first and third quartiles, respectively, while IQR is the interquartile range) are excluded. A smoothing curve is then fitted by a re-descending M estimator with Tukey’s biweight function^6^. This enables the fit to be resistant to noise arising from species heterogeneity and low coverage. GRiD values represent the coverage ratio of the peak and trough of the curve, in which case, GRiD values are directly proportional to growth rate.

Generally, coverage information derived from a sample containing multiple diverse strains can be prone to noise, which will invariably have an impact on accurate growth estimates. In other words, core regions of genomes will have unusually high coverage compared to accessory regions. Similarly, microbes present at ultra-low coverage can be prone to noise due to a wider coverage distribution resulting from contigs with an average coverage of zero. To circumvent these limitations, GRiD incorporates a refinement step. Here, for the peak coverage value, the lowest point of expected variation of the mean value is chosen. Likewise, the upper point of variance of the trough mean is selected for the trough value (Supplementary Fig. 1C). The resulting growth value is called “GRiD_refined”. This refinement step markedly improves the reproducibility of GRiD predictions (Supplementary Fig. 1D). Additionally, to account for uncertainty arising from contig reordering, GRiD performs a subsampling step twice by randomly extracting 85% of reads that mapped to a genome. GRiD then calculates and outputs a 95% confidence interval from all three growth values (i.e., including GRiD_refined). Finally, ambiguous reads (i.e., reads mapping to multiple genomes) can result in erroneous estimates. GRiD outputs a metric called “species heterogeneity” which measures the contribution of ambiguous reads to growth estimate. Here, species heterogeneity = (1 – *r*/*u*), where *r* = GRiD_refined, and *u* = GRiD_unrefined (i.e., growth rate calculated directly from the means of the peak and trough).

Additionally, since metagenomic bins can be contaminated with contigs from other genomes, which may impact growth predictions especially in samples where contaminating contigs contain high coverage, GRiD estimates can be quality-checked, whenever possible, using the coverage information of chromosome initiator replication gene (*dnaA*) and deletion-induced filamentation (*dif*) sequences across the genome. *dnaA* is usually located close to the *ori* (Supplementary Fig. 1E), whereas replication terminates at or near *dif* sequences^7–9^. Therefore, in rapidly dividing cells, the coverage values for the peak and *dnaA* should be similar. Likewise, the coverage values for *dif* and *ter* should be identical. GRiD output includes both *dnaA/ori* and *ter/dif* ratios. Growth rate estimates are most likely accurate when *dnaA/ori* and *ter/dif* coverage ratios approach one, and species heterogeneity is low (<0.3) (Supplementary Fig. 2A). Altogether, GRiD outputs seven values for every sample; (i) GRiD refined, (ii) 95% confidence interval, (iii) GRiD unrefined, (iv) species heterogeneity, (v) coverage, (vi) *dnaA/or*i ratio, and (vii) *ter/dif* ratio.

### GRiD-MG

For multiplex screening of the identified bacterial population in a given sample, GRiD-MG maps metagenomics reads to a custom GRiD-MG database consisting of 32,819 bacterial genomes (Fig. 3a). This database contains one representative genome per specie. In additional, the GRiD-MG database can be easily updated using metagenomic bins or newly sequenced genomes. Depending on user preferences, reads mapping to multiple genomes are reassigned using Pathoscope 2.0^18^ and the extent to which reads are reassigned is defined using the “theta-prior” option. In addition, a user can specify a coverage cutoff greater than 0.2× in which case, genomes with coverage below the cutoff are discarded. For coverage cutoff <1×, only genomes with 90 fragments/Mbp or less are included in the analysis since increased fragmentation results in inaccurate results at ultra-low coverage (Supplementary Figure 2B). We included additional stringency to the algorithm to reduce the likelihood of outputting false growth estimates. First, upon coverage calculation using a 10kb window as described above, genomes with coverage medians <0.15 are considered as non-replicating. Second, GRiD values greater than 10 are discarded as this may be due to a high coverage of a contaminant contig present in a metagenomic bin or genome in a given sample. Third, GRiD values greater than 3 for genomes with sizes less than 4 Mb are discarded. This is because GRiD values greater than 2 indicate the presence of multi-fork replication machinery which has mostly been identified in genomes with sizes >4Mb^20–22^. Two output files are generated per sample; a text file of genomes and their respective GRiD scores, and a pdf file displaying heatmap of growth values with hierarchical clustering (Fig. 3a). Text output for all samples can be merged into a single matrix file.

### *dnaA* and *dif* database

To determine the coverage of *dnaA* and *dif* sequences, we built a database comprising of 217 bacterial *dnaA* sequences that were obtained from NCBI, and 714 *dif* sequences retrieved from the Database of Bacterial Replication Terminus (http://www.g-language.org/data/repter/db.html). We then searched for homologs within a bin or genome using BLAST^23^ (-evalue 0.05) and the topmost hit was selected.

### GRiD and iRep growth calculation from in vitro *S. epidermidis* and *C. simulans*

*S. epidermidis* (NIHLM023) and *C. simulans* (Wattiau) were grown in Tryptic Soy Broth at 37 °C. During cultivation, samples were harvested at five different time points between early and mid-exponential growth phase for DNA extraction and whole genome sequencing. We calculated GRiD and iRep using draft genomes of *S. epidermidis* (NIHLM023; 87 contigs) and *C. simulans* (strain 1B08; 66 contigs).

### GRiD and iRep growth calculation for CPR genomes

We retrieved previously reported iRep data calculated from 12 samples using 99 CPR genomes^5^. However, we restricted GRiD analysis to bacteria with draft genomes.

### Metagenomic binning of skin dataset

We retrieved 698 metagenomic shotgun skin samples from our previous work^10^. Some samples (*n* = 594) in this dataset were derived from longitudinal sampling of 12 individuals at three different time points with sampling intervals of 10–30 months between time points 1 and 2 (T1 vs T2), and 5–10 weeks between time points 2 and 3 (T2 vs T3). We began by concatenating all samples and assembling sequence reads into contigs and scaffolds using MEGAHIT^24^. We initially chose MEGAHIT due to its capability for handling large data, low memory requirements, and short run time. We discarded contigs shorter than 1 kb, mapped each individual sample back to the contigs catalogue using bowtie2^25^, and extracted unmapped reads (Supplementary Fig. 4). Next, we concatenated unmapped reads and re-assembled using SPAdes (--meta)^26^. We chose SPAdes due to its ability to produce scaffolds from contigs. The newly extracted contigs/scaffolds were merged with the previous catalogue. We grouped our contigs/scaffolds into genome bins using MetaBAT (--sensitive, -m 1500)^12^, which resulted in 556 bins, and subsequently utilized MEGAN^27^ for taxonomic identification of contigs/scaffolds present within each bin. We excluded 22 bins that were of non-microbial origin and further evaluated the quality of each bin and marker lineage using CheckM^28^. For stringent annotation, we required that ≥65% of contigs/scaffolds present in a bin are assigned to the lowest level taxonomy; the sole exception being the kingdom-level taxonomy where our requirement was 40%.

### Determining the effect of genome fragmentation and completeness on GRiD

We selected 12 bacteria bins (≥95% completeness and ≤5% contamination) with varying degree of fragmentation ranging from 55 to 202 fragments/Mbp to determine the role of fragmentation on GRiD scores. For each bin, we used the sample producing the highest bin coverage. Reads mapping to each bin was subsampled to 0.2×, 0.4×, and 1× coverage for GRiD analysis. We concluded that bins with 90 or more fragments/Mbp are unsuitable for GRiD analysis at coverage levels below 1× (Supplementary Fig. 2B).

We used a bin having 89 fragments/Mbp, which was at the boundary for our accuracy cutoff at ultra-low coverage, to evaluate the impact of genome completeness on GRiD output. We subsampled contigs within each bin to attain the desired level of completeness prior to GRiD analysis. This subsampling step was repeated 10 times.

### GRiD estimate of rare bacteria

We chose only high-quality bins (i.e., ≥75% completeness and ≤5% contamination based on single copy marker genes) for analysis. We defined a bin as rare if it was present in fewer than 3% of samples. Three bins (bins 257, 443, 481) met this threshold and were considered uncharacterized since they were unable to be annotated to the species level using either our 65% cutoff as mentioned above, or single copy marker gene lineage using CheckM^28^. Since these bins had >90 fragments/Mbp, we calculated GRiD in samples having bin coverage ≥1×.

### GRiD calculations from psoriasis skin dataset

We retrieved 73 metagenomic samples from Tett et al.^13^, which was a collection of skin samples from individuals with psoriasis. We obtained the species composition using Pathoscope 2.0^18^ and selected only species that were present in at least five samples (*n* = 35).

### GRiD estimate of *Bdellovibrio* species

From a previous genome construction effort using >1500 samples^16^, we retrieved samples where species of *Bdellovibrio* were reconstructed (*n* = 22). Of these, 15 samples were from marine environments, 3 from sludge, 2 from freshwater, and 1 each from groundwater and hydrocarbon environment. We calculated a total of 134 replication rates from these environments. We used Pathoscope 2.0^18^ to determine the relative abundance of microbes.

### Generation of mock reads for GRiD-MG

We generated mock read for *Lactobacillus gasseri*, *Parabacteroides distasonis*, *Staphylococcus aureus, Staphylococcus epidermidis*, and *Campylobacter upsaliensis* using wgsim package implemented in samtools^29^. Each genome was split into 100kb fragments and varying amount of reads was generated for each fragment in order to achieve differential coverage across the genome, and thus, mimic a replicating genome.

### Growth antagonism of skin bacteria

We applied GRiD-MG to 698 metagenomic skin dataset collected from different skin sites. These samples were earlier classified into four groups based on their site characteristics as either dry, moist, oily, or foot sites^10^. We subsampled 1 million reads for samples containing over a million reads. We used a coverage cutoff of 0.2× thereby discarding growth rate for genomes having <90 fragments/Mbp. We also did not utilize the Pathoscope reads reassignment option. This resulted in growth estimates for 1,756 bacterial species. For each site, correlation analyses were conducted for genomes present in >40% of samples. We identified biosynthetic gene clusters in *Staphylococcus pasteuri* strains using antiSMASH^30^

### Code availability

The GRiD algorithm is available at https://github.com/ohlab/GRiD.

## Electronic supplementary material

Supplementary Information

Reporting Summary

Source Data

## Data Availability

Sequence reads generated for *S. epidermidis* and *C. simulans* in vitro growth analyses have been deposited in SRA with accession/identification number SRP151711. The accession numbers are available from NCBI for longitudinal skin dataset (BioProject PRJNA46333), groundwater filtrates (BioProject PRJNA268031), and psoriasis patients (BioProject PRJNA281366). For *Bdellovibrio* (BioProject PRJNA348753) analysis, the following Sequence Reads Archive accessions were used; SRR948155, SRR948284, SRR2043728, SRR636581, ERR599000, ERR594331, ERR594318, ERR599136, ERR599142, ERR594299, ERR594326, ERR594335, ERR599038, ERR599044, ERR594348, ERR594349, ERR594311, ERR594294, ERR594308, SRR1506988, SRR1506983, SRR1506986. CPR genomes (BioProject PRJNA273161) are available from NCBI GenBank. A reporting summary for this Article is available as a Supplementary Information file. The source data underlying Fig. 1–3, and Supplementary Figs 1–3 are provided as a Source Data file. All relevant data are available upon request.
