# Supplementary material for: High throughput in situ metagenomic measurement of bacterial replication at ultra-low sequencing coverage
Source: Nat Commun. 2018 Nov 23;9:4956. doi: 10.1038/s41467-018-07240-8 (PMC6251912; doi:10.1038/s41467-018-07240-8)
Supplement: Supplementary file 1 — Supplementary Information [file 41467_2018_7240_MOESM1_ESM.pdf]

High throughput in-situ metagenomic measurement of bacterial replication at ultra-low sequencing coverage

Emiola and Oh

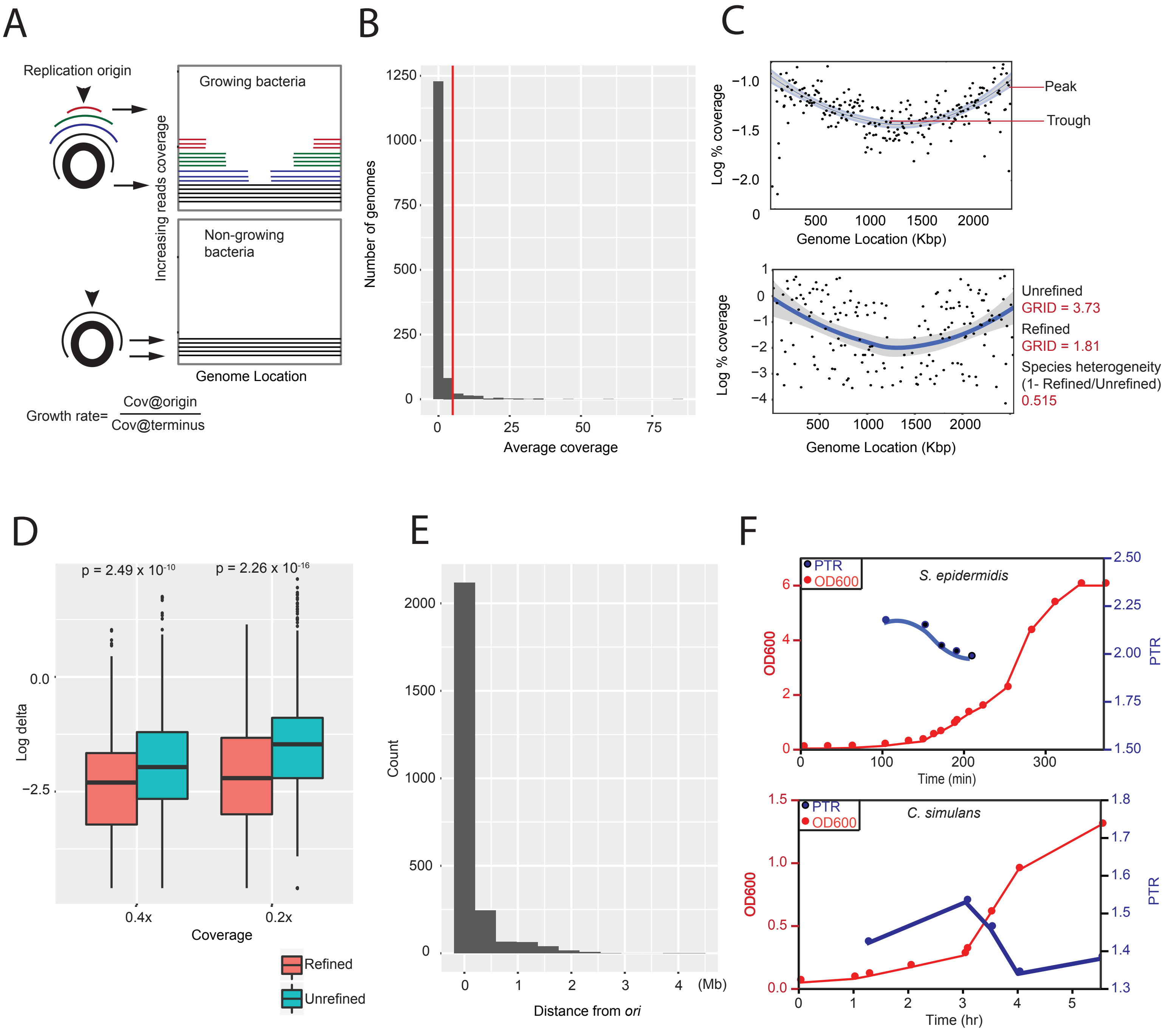

Supplementary Figure 1: GRiD benchmark

(A) Growing bacteria have higher read coverage in regions close to the origin of replication (ori) compared to the terminus (ter) region. Growth rate can be measured as the ratio of coverage at the ori and ter regions. (B) Average coverage of genomes calculated from a metagenomic skin dataset (n = 698) with median read count of 17.9 million reads per sample10. The red vertical line represents a coverage cutoff of 5x which is required by iRep. (C) To minimize the level of noise during GRiD estimation, GRiD utilizes the lowest point of expected variance of the mean for the peak value, while the upper point of the variance of the trough mean is selected. The lower figure shows GRiD calculations of S. epidermidis in a skin sample with or without refinement. Overestimation of growth rate could occur without refinement. (D) Reproducibility of S. epidermidis GRiD estimates from a skin dataset after subsampling in the presence and absence of refinement. GRiD estimates are significantly ($p < 0.001$, Wilcoxon rank-sum test) more reproducible when refinement is included. (E) Barplot showing the distance of dnaA from ori in 2561 bacterial genomes obtained from the Database of Replication Origins (doriC) (http://tubic.tju.edu.cn/doric/index.php). (F) In vitro growth curve of S. epidermidis and C. simulans obtained from pure cultures and the corresponding PTR. Both microbes had an exponential doubling time of 30 min. Source data are provided as a Source Data file.

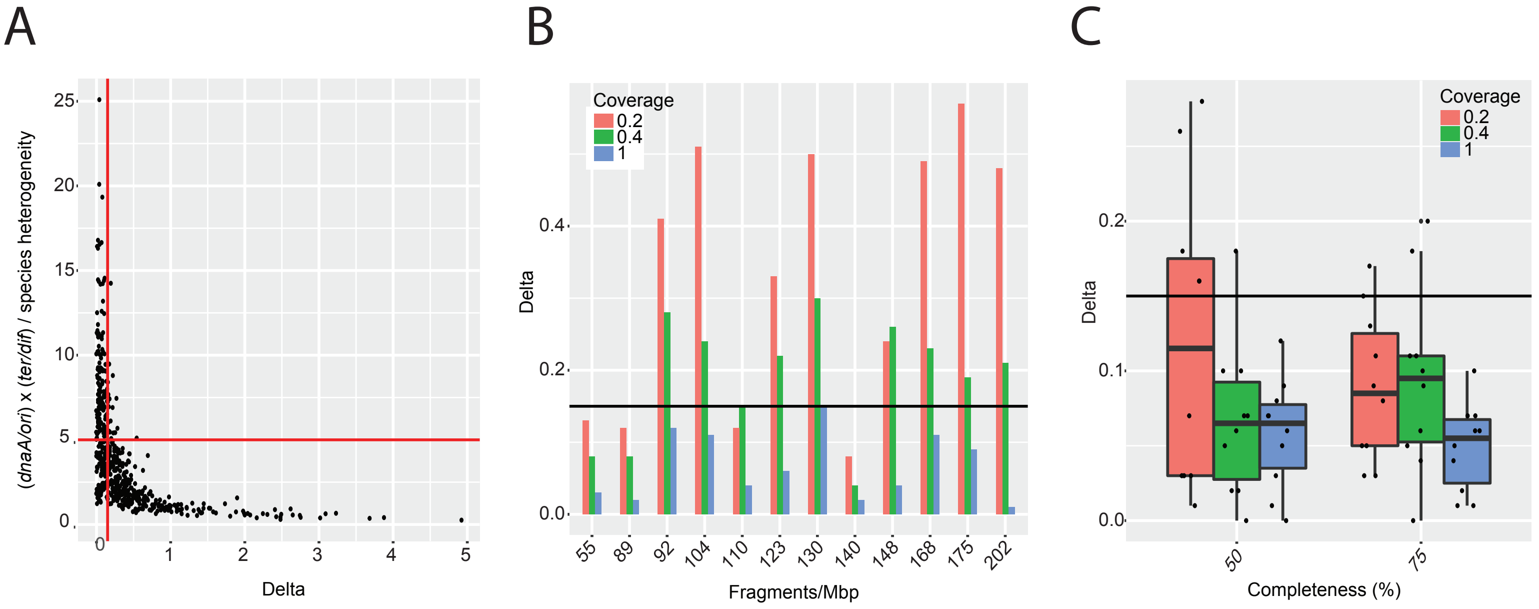

Supplementary Figure 2: Assessment of GRiD parameters

(A) The combined role of dnaA coverage, dif coverage, and species heterogeneity on GRiD accuracy. PTR was initially calculated for S. epidermidis using a closed circular reference genome from a skin dataset, and then, GRiD was calculated using the same reference genome, but fragmented into 100 Kb fragments and reshuffled. The differences in growth estimates (delta) between PTR and GRiD (x-axis) are displayed as a factor of dnaA coverage, dif coverage, and species heterogeneity (y-axis). The red vertical line represents a delta cutoff of 0.15 which we considered as the threshold for high accuracy while the horizontal line is the y-axis cutoff for high accuracy. (B) The effect of genome fragmentation on GRiD reproducibility using 12 high quality bacterial bins (≥ 95% completeness and ≤ 5% contamination) with varying degree of fragmentation ranging from 55 to 202 fragments/Mbp. The horizontal line represents a delta cutoff of 0.15. (C) The effect of genome completeness on GRiD reproducibility. A genome bin with 89 fragments/Mbp, which is at the boundary for accuracy cutoff at ultra-low coverage as shown in ‘B’ above, was randomly subsampled prior to GRiD analysis. This subsampling step was conducted 10 times. The horizontal line represents a delta cutoff of 0.15. Source data are provided as a Source Data file.

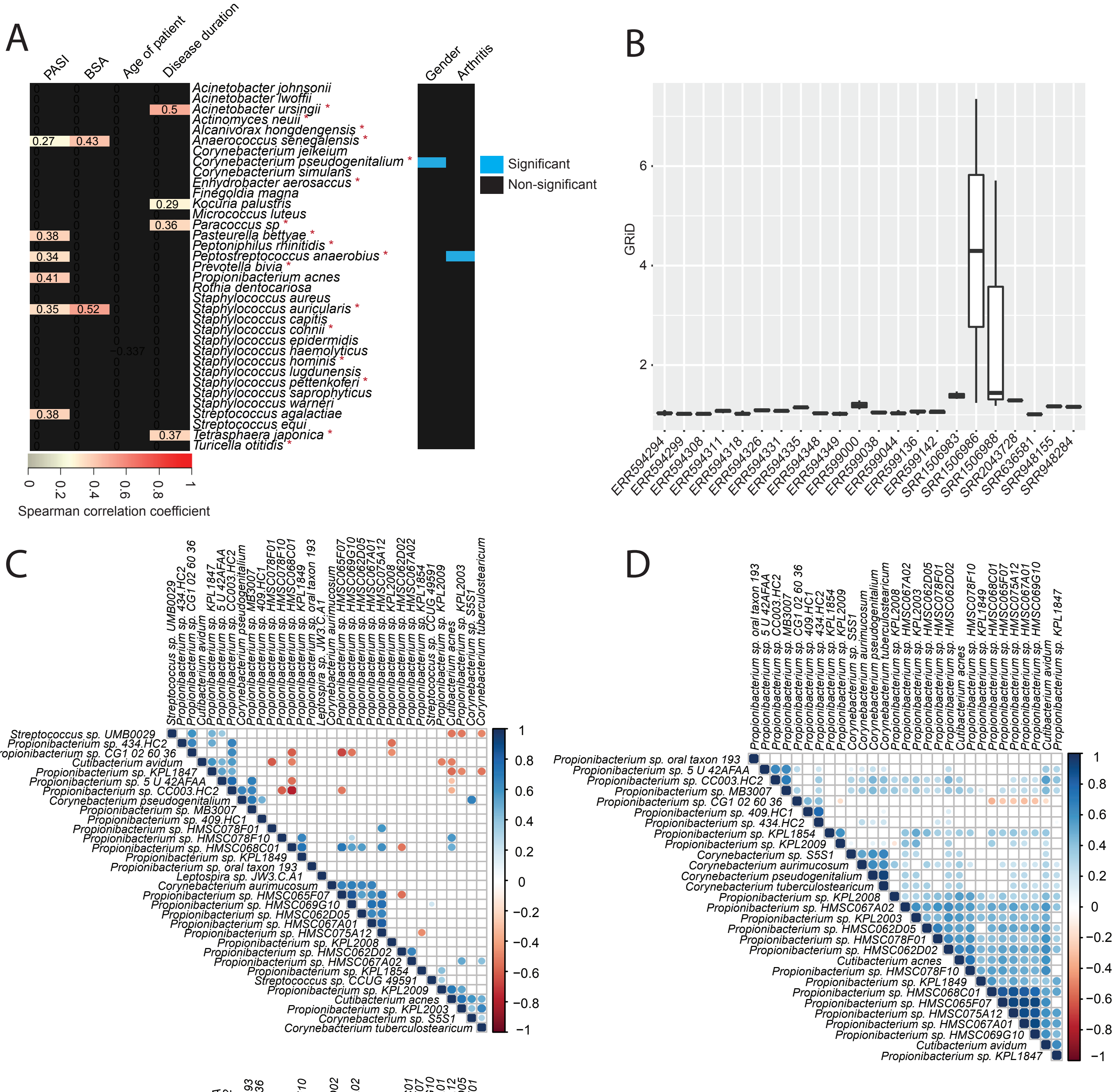

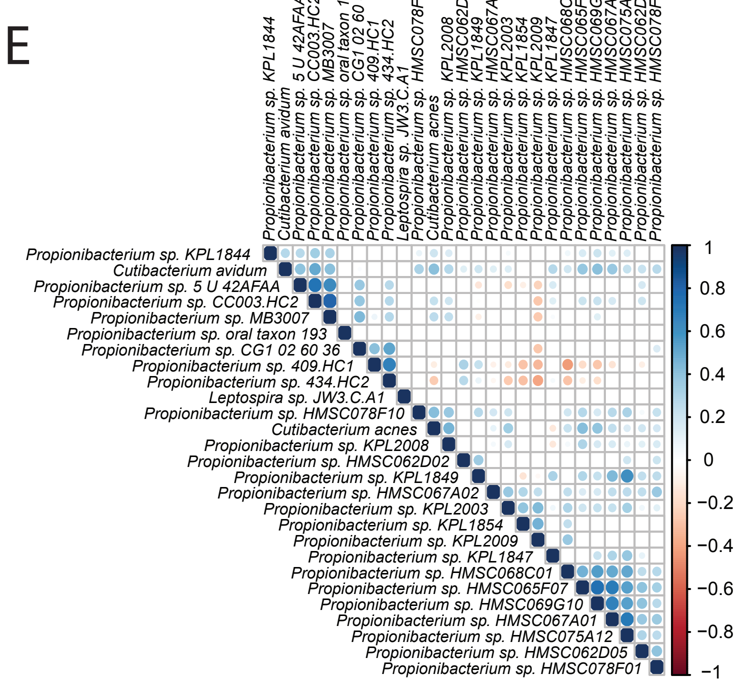

Supplementary Figure 3: GRiD analysis from metagenomic dataset

(A) Inter-individual bacterial growth differences and association with psoriasis patient characteristics. Statistical differences between population groups were determined using the Wilcoxon rank-sum test. (B) GRiD values of Bdellovibrio species in each environmental sample. (C, D, E) Growth rate correlation between bacterial species in dry (C), moist (D) and oily (E) sites from a skin metagenomic dataset. Blue and red circles indicate positive and negative Spearman correlation respectively. Larger circles and darker colors indicate a higher correlation. Source data are provided as a Source Data file.

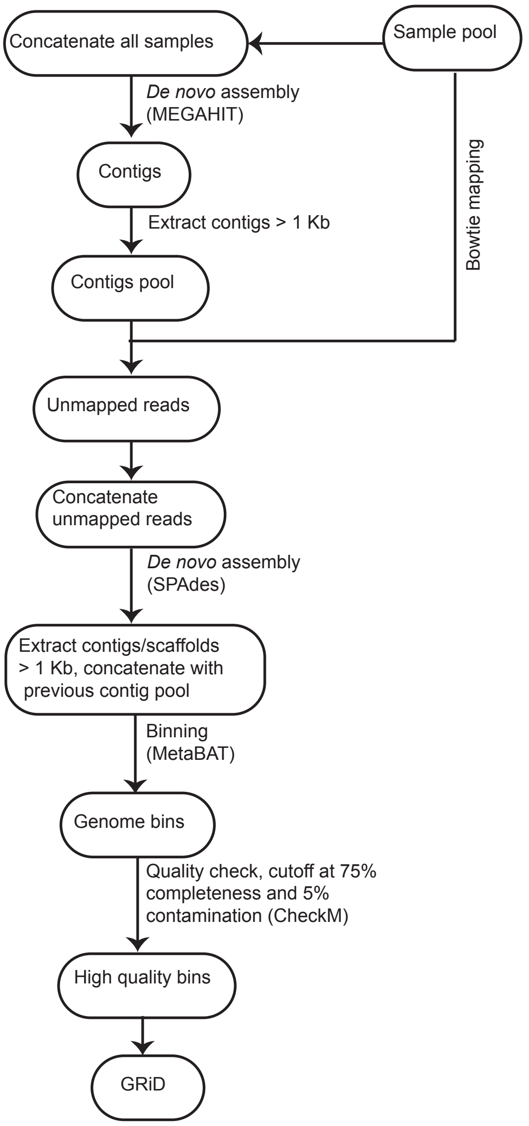

Supplementary Figure 4: Flowchart for the identification of uncultivated bacteria prior to GRiD analysis.
