# Supplementary material for: High throughput in situ metagenomic measurement of bacterial replication at ultra-low sequencing coverage
Source: Nat Commun. 2018 Nov 23;9:4956. doi: 10.1038/s41467-018-07240-8 (PMC6251912; doi:10.1038/s41467-018-07240-8)
Supplement: Supplementary file 2 — Reporting Summary [file 41467_2018_7240_MOESM2_ESM.pdf]

nature research

Corresponding author(s): Julia Oh

Reporting Summary

Nature Research wishes to improve the reproducibility of the work that we publish. This form provides structure for consistency and transparency in reporting. For further information on Nature Research policies, see Authors & Referees and the Editorial Policy Checklist.

Statistical parameters

When statistical analyses are reported, confirm that the following items are present in the relevant location (e.g. figure legend, table legend, main text, or Methods section).

n/a	Confirmed	
☐	☒	The exact sample size (*n*) for each experimental group/condition, given as a discrete number and unit of measurement
☐	☒	An indication of whether measurements were taken from distinct samples or whether the same sample was measured repeatedly
☐	☒	The statistical test(s) used AND whether they are one- or two-sided<br>*Only common tests should be described solely by name; describe more complex techniques in the Methods section.*
☒	☐	A description of all covariates tested
☒	☐	A description of any assumptions or corrections, such as tests of normality and adjustment for multiple comparisons
☐	☒	A full description of the statistics including central tendency (e.g. means) or other basic estimates (e.g. regression coefficient) AND variation (e.g. standard deviation) or associated estimates of uncertainty (e.g. confidence intervals)
☒	☐	For null hypothesis testing, the test statistic (e.g. *F*, *t*, *r*) with confidence intervals, effect sizes, degrees of freedom and *P* value noted<br>*Give P values as exact values whenever suitable.*
☒	☐	For Bayesian analysis, information on the choice of priors and Markov chain Monte Carlo settings
☒	☐	For hierarchical and complex designs, identification of the appropriate level for tests and full reporting of outcomes
☒	☐	Estimates of effect sizes (e.g. Cohen's *d*, Pearson's *r*), indicating how they were calculated
☒	☐	Clearly defined error bars<br>*State explicitly what error bars represent (e.g. SD, SE, CI)*

Our web collection on statistics for biologists may be useful.

Software and code

Policy information about availability of computer code

Data collection	NA
Data analysis	GRiD algorithm was used for analysis (https://github.com/ohlab/GRiD)

For manuscripts utilizing custom algorithms or software that are central to the research but not yet described in published literature, software must be made available to editors/reviewers upon request. We strongly encourage code deposition in a community repository (e.g. GitHub). See the Nature Research guidelines for submitting code & software for further information.

Data

Policy information about availability of data

All manuscripts must include a data availability statement. This statement should provide the following information, where applicable:

- Accession codes, unique identifiers, or web links for publicly available datasets
- A list of figures that have associated raw data
- A description of any restrictions on data availability

Sequence reads generated for S. epidermidis and C. simulans in vitro growth analyses have been deposited in SRA with accession/identification number SRP151711. The accession numbers are available from NCBI for longitudinal skin dataset, (BioProject PRJNA46333), groundwater filtrates (BioProject PRJNA268031), and psoriasis patients (BioProject PRJNA281366). For Bdellovibrio (BioProject PRJNA348753) analysis, the following Sequence Reads Archive accessions were used;

SRR948155, SRR948284, SRR2043728, SRR636581, ERR599000, ERR594331, ERR594318, ERR599136, ERR599142, ERR594299, ERR594326, ERR594335, ERR599038, ERR599044, ERR594348, ERR594349, ERR594311, ERR594294, ERR594308, SRR1506988, SRR1506983, SRR1506986. CPR genomes (BioProject PRJNA273161) are available from NCBI GenBank

Field-specific reporting

Please select the best fit for your research. If you are not sure, read the appropriate sections before making your selection.

[x] Life sciences   [ ] Behavioural & social sciences   [ ] Ecological, evolutionary & environmental sciences

For a reference copy of the document with all sections, see nature.com/authors/policies/ReportingSummary-flat.pdf

Life sciences study design

All studies must disclose on these points even when the disclosure is negative.

Sample size	GRiD algorithm was tested using 4 different metagenomics datasets from human and environmental microbiomes
Data exclusions	No data exclusion
Replication	We performed computational analysis thus no replication was needed
Randomization	NA
Blinding	NA

Reporting for specific materials, systems and methods

Materials & experimental systems

n/a	Involved in the study
[x]	[ ] Unique biological materials
[x]	[ ] Antibodies
[x]	[ ] Eukaryotic cell lines
[x]	[ ] Palaeontology
[x]	[ ] Animals and other organisms
[x]	[ ] Human research participants

Methods

n/a	Involved in the study
[x]	[ ] ChIP-seq
[x]	[ ] Flow cytometry
[x]	[ ] MRI-based neuroimaging
